# CyclinD1 protein expressed in pterygia is associated with β-catenin protein localization

**Published:** 2010-12-15

**Authors:** Jai-Nien Tung, Chun-Chi Chiang, Yi-Yu Tsai, Ying-Yi Chou, Kun-Tu Yeh, Huei Lee, Ya-Wen Cheng

**Affiliations:** 1 Institute of Medicine, Chung Shan Medical University, Taichung, Taiwan; 2 Department of Neurosurgery, Tungs’ Taichung MetroHarbor Hospital, Taichung, Taiwan; 3 Department of Ophthalmology, China Medical University Hospital, Taichung, Taiwan; 4 Graduate School of Dentistry, Chung Shan Medical University, Taichung, Taiwan; 5 Department of Pathology, Changhua Christian Hospital, Changhua, Taiwan; 6 Institute of Medical & Molecular Toxicology, Chung Shan Medical University, Taichung, Taiwan; 7 Department of Medical Research, Chung Shan Medical University Hospital, Taichung, Taiwan; 8 Division of Environmental Health and Occupational Medicine, National Health Research Institutes, Zhunan, Miaoli County, Taiwan, ROC

## Abstract

**Background:**

The Wnt (Wg/Wnt) signaling cascade plays an important role in tumorigenesis. Our previous report indicated that aberrant localization of β-catenin proteins was a feature of pterygia. Therefore, this study aimed to analyze the association of β-catenin protein and expression of a downstream gene, cyclin D1, in pterygial tissues.

**Methods:**

Using immunohistochemistry, β-catenin and cyclin D1 protein expression was studied, in 150 pterygial specimens and 30 normal conjunctivas.

**Results:**

Seventy-three (48.7%) and 60 (40.0%) pterygial specimens tested positive for β-catenin and cyclin D1 protein expression, respectively. Cyclin D1protein expression was significantly higher in β-catenin-nuclear/cytoplasmic positive groups than in β-catenin membrane positive and negative groups (p<0.0001). In addition, cyclin D1 expression was significantly higher in the fleshy group than in the atrophic and intermediate groups (p=0.006).

**Conclusions:**

Our study demonstrated that β-catenin expressed in nuclei/cytoplasm increases cyclinD1 protein expression, which invokes pterygial cell proliferation.

## Introduction

Pterygium has long been considered a degenerative disease. However, the p53 protein was found to be abnormally expressed in epithelium; consequently, pterygium is now considered to be a UV exposure-related uncontrolled cell proliferation that is similar to a tumor [1-7].

Several adhesion molecules, including cadherin, cell-cell adhesion molecules (CAMs), selectin, and integrin, maintain tissue architecture [8]. E-cadherin (120 kDa; chromosome 16q), a transmembrane protein, plays a role in organ morphogenesis, tissue formation, and proper development during embryogenesis [9]. The extracellular domain of E-cadherin connects neighboring cells that share adherent junctions through calcium-dependent homophilic interactions [9]. The cytoplasmic portion of E-cadherin links the cytoskeleton via the proteins α-catenin, β-catenin, and p120ctm [10]. β-catenin not only contributes to cellular adhesion but also is a central component in the Wingless/Wnt (Wg/Wnt) signaling cascade, an important control system for axis determination and organogenesis during early development [11,12].

In the absence of a mitotic signal, free cytoplasmic β-catenin is degraded by the ubiquitin-proteasome system following phosphorylation by the protein complex, which is composed of adenomatous polyposis coli (APC) tumor suppresser protein, Axin, and serine threonine glycogen synthetase kinase (GSK-3β) [13]. This mechanism ensures a low level of free cytoplasmic β-catenin. However, with the appearance of mitotic signals (e.g., Wnt protein) or with reduced expression of E-cadherin, the levels of free β-catenin in the cytoplasm begin to increase. The binding of the Wnt protein to its cognate frizzled receptor leads to activation of the dishevelled (Dsh) protein, which then down-regulates the APC-Axin- GSK-3β protein complex [14]. Hence, cytoplasmic β-catenin evades degradation and accumulates in the cytoplasm [15,16].

In addition, reduced expression of E-cadherin leads to both the decomposition of the E-cadherin-catenin complex and an increase in free cytoplasmic β-catenin. E-cadherin also mediates epithelial cellular adhesion. When the free cytoplasmic β-catenin increases, it eventually translocates into the nucleus and binds with transcription factors LEF (Lymphoid Enhancer Factor) and TCF (T Cell Factor), resulting in the activation of target genes [17,18] such as cyclin D1 and c-myc, are responsible for cell proliferation and neoplastic transformation [19,20]. By this process, activation of cyclinD1 by β-catenin contributes to epithelial differentiation.

Cyclin D1 is a well known cell cycle control gene that promotes cell cycle progression through the G_1_-phase by forming active holoenzymes with CDK (cyclin-dependent kinase) 4 and CDK6 and leads to phosphorylation of pRb (retinoblastoma protein) [21]. Phosphorylation causes pRb to release the E2F transcription factor, which can then activate genes essential for progression through the G_1–S_ transition and S-phase [22].

Our previous report indicated that promoter hypermethylation of E-cadherin may be involved in the reduction of E-cadherin protein expression in pterygium [23]. We also provided evidence to show that the aberrant protein localization of β-catenin was detected in pterygium [23]. As a result of our findings, we suggest that changes in β-catenin signaling indeed play a causative role in the development of pterygium.

In the present study, we hypothesized that β-catenin expressed in nuclei/cytoplasm could increase cyclin D1 protein expression to involve the formation of pterygia. To test these hypotheses, we analyzed both the expression of β-catenin and cyclin D1 in pterygium and the relationship between β-catenin protein localization and cyclin D1 protein expression.

## Methods

### Study subjects

Pterygial samples were harvested from 150 patients undergoing pterygium surgery and the patients were asked to submit a written informed consent approved by the Institutional Review Board. Patients in whom the apex of the pterygium had invaded the cornea by more than 1 mm were included in this study. The pterygia were classified into grades 1, 2, or 3 based on slit-lamp biomicroscopy evaluation. Grade 1 (“atrophic”) had clearly visible episcleral vessels under the body of the pterygium; grade 2 (“intermediate”) had partially visible episcleral vessels under the body of the pterygium; grade 3 (“fleshy”) had totally obscured episcleral vessels underlying the body of the pterygium. The controls included normal conjunctival samples collected from the superior conjunctiva of 15 patients and the medial conjunctiva of 15 patients without pterygium and pinguecula; all patients were undergoing cataract or vitreoretinal surgery. There were 87 males and 63 females in the pterygium group (age range=55 −82 years, means=65.7 years), and there were 15 males and 15 females in the control group (age range=55–75 years, mean=62.8 years). Normal conjunctival samples were collected from bulbar conjunctivas. All pterygial specimens came from primary pterygia. All specimens were fixed in formalin and paraffin embedded.

### Immunohistochemistry

All sections were deparaffinized in xylene, sequentially rehydrated through serial dilutions of alcohol and washed in phosphate-buffered saline. Sections used for β-catenin and cyclin D1, detection were heated in a microwave oven twice for 5 min in citrate buffer (pH 6.0). Mouse anti-β-catenin and a cyclin D1 monoclonal antibody (at a dilution of 1:200; Santa Cruz, Santa Cruz, CA) were used as the primary antibodies. The visualization was performed by DAB (3,3′-diaminbenzidine chromogen) using the DAKO LSAB/HRP kit [24]. Negative controls that did not include the primary antibodies were also prepared. Conjunctiva tissues and lung epithelial cells were used as the positive control. The results were evaluated independently by three observers and were scored for the percentage of positive expression. For cyclin D1 protein, which was expressed only in the nuclei, we counted the positive rate of nuclear expression in all epithelial cells. For the β-catenin, which was expressed in the membrane, cytoplasm, and nuclei, we separated the expression site into two groups, membrane expression and cytoplasm/nuclear expression. In this study, three of 150 patients have positive immunostaining in both membrane and cytoplasm. We placed them in the nuclei/cytoplasm groups because in the membrane group, the beta-catenin was expressed only in the membrane. Scores were indicated as the following: score 0, no positive staining; score +, from 1% to 10%; score ++, from 11% to 50%; and score +++, more than 50% positive cells. In this study, scores of +, ++, and +++ were considered to represent positive immunostaining, and a score of 0 was classified as negative immunostaining.

### Statistical analysis

A statistical analysis was performed using the SPSS statistical software program (SPSS Inc., Chicago, IL). The Fisher’s exact test and χ^2^ test were applied for statistical analysis. A p<0.05 was considered to be statistically significant.

## Results

### β-catenin and cyclin D1 protein expression in pterygium

**Figure 1:**
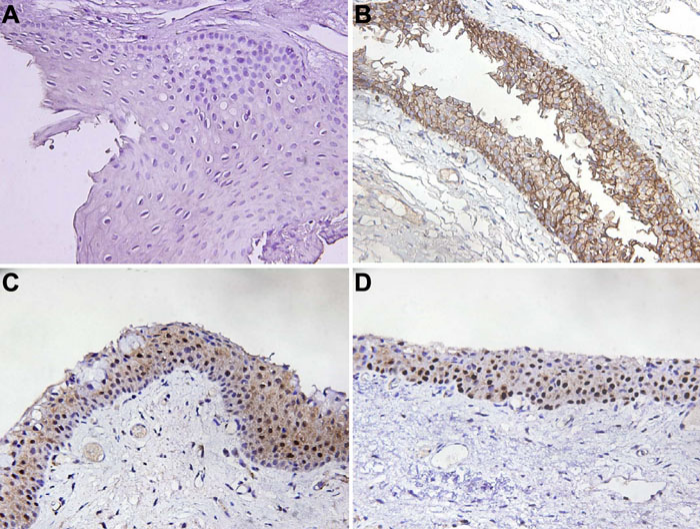
Representative immunostaining results for β-catenin and cyclin D1. **A**: the negative control has the first antibody replaced with IgG. **B**: β-catenin protein expression detected in the membrane (400×), **C**, aberrant localization of β-catenin in the cytoplasm/nuclei (400×), and **D**, cyclin D1 protein expression in nuclei (400×).

When the free cytoplasmic β-catenin increases, it ultimately translocates into the nucleus, resulting in the activation of cyclin D1 and c-myc, which are responsible for cell proliferation and neoplastic transformation [19,20]. We further analyzed the correlation of β-catenin and cyclinD1 protein expression in pterygium. In the pterygium group, the β-catenin proteins were detected in 73 (48.7%) patients (Table 1). Additionally, as shown in Figure 1, aberrant protein localization of β-catenin was also detected in pterygia. In 73 β-catenin positive patients, 60.3% (44 of 73) showed membrane localization and 39.7% (29 of 73) showed a cytoplasmic or nuclear localization (Figure 1A). In the normal conjunctiva group, all specimens were positive for immunostaining in the membrane. These results were similar to those of our previous report [23]. In the pterygium group, cyclin D1 proteins were detected in 60 (40.0%) patients (Table 1). Cyclin D1 was positive for immunostaining in the nuclei (Figure 1B). In the normal conjunctiva group, only 2 of 30 specimens were positive for immunostaining in the nuclei.

**Table 1:** β-catenin and cyclin D1 protein expression in pterygial and control conjunctiva analyzed by immunohistochemistry.

** **	**Pterygium**		**Control**	
**Protein**	**N**	**%**	**N**	**%**
**β-catenin**				
Negative	77	51.3	0	0
**Positive**				
Membrane	44	29.3	30	100
Nuclei/Cytoplasm	29	19.4	0	0
**Cyclin D1**				
Negative	90	60.0	28	93.3
Positive	60	40.0	2	6.7

β-catenin analyzed in this study is based on prior pterygium samples [23].

### Correlation of β-catenin and cyclin D1 protein expression

To understand the relationship between the β-catenin and cyclinD1 protein expression, the correlations of β-catenin localization and cyclin D1 protein expression were analyzed (Table 2). As shown in Table 2, in the 77 cases of β-catenin negative expression, 74.0% (57 of 77) had cyclin D1 negative expression and 26.0% (20 of 77) had positive nuclear expression. In the 44 cases of β-catenin membrane expression, 68.2% (30 of 44) had cyclin D1 negative expression, and 31.8% (14 of 44) had positive nuclear expression. Additionally, in the 29 cases of β-catenin nuclear/cytoplasmic expression, 10.3% (3 of 29) had cyclin D1 negative expression and 89.7% (26 of 29) had positive nuclear expression. The cyclin D1 protein nuclear expression in the β-catenin group showing aberrant protein localization was significantly higher than in the negative and membrane groups (p<0.0001).

**Table 2:** Relationship of β-catenin protein localization and cyclin D1 protein expression in pterygia.

** **	**Cyclin D1**				** **
** Protein**	**Negative % (n=90)**		**Positive % (n=60)**		**p value**
**β-catenin**					
Negative (n=77)	57	(74.0)	20	(26.0)	** **
**Positive**					
Membrane (n=44)	30	(68.2)	14	(31.8)	** **
Nuclei/Cytoplasm (n=29)	3	(10.3)	26	(89.7)	<0.0001

### Cyclin D1 protein expression correlated with the grade of pterygium

To understand whether the cyclin D1 protein expression was correlated with the pterygium development, the correlation of cyclin D1 protein expression and the grade of pterygium was analyzed. As showed in Table 3, expression of cyclin D1 protein in atrophic, intermediate, and fleshy groups were, respectively, 34.6% (18 of 52), 31.1% (19 of 42) and 56.8% (21 of 37; p=0.006). The cyclin D1 protein expression in the fleshy group was significantly higher than in the other two groups (p=0.006). No significant differences were found between cyclin D1 protein expression and gender (p=0.38). Therefore, we suggested that the cyclin D1 protein may be involved in pterygium progression.

**Table 3:** Correlation of cyclin D1 protein expression and clinical parameters of pterygium.

** **	**Cyclin D1**		** **
**Parameters**	**Negative (n=90)**	**Positive (n=60)**	**p value**
**Gender**			
Female (n=63)	27	11	** **
Male (n=87)	26	18	0.238
**Type**			
Atrophic (n=53)	34	19	** **
Intermediate (n=62)	43	19	** **
Fleshy (n=35)	13	22	0.006

## Discussion

Pterygium, previously considered a degenerative process of the corneal limbus, pterygium is characterized by the invasion of a fleshy triangle of conjunctival tissue onto the cornea. Shimmura et al. demonstrated an increased activity of telomerase in pterygial epithelial cells, indicating their hyperproliferative nature [25]. Satoru et al. [26] reported that increased expression of Ki67, a cell proliferation marker, was noted in pterygia. We assume that it is the proliferative capacities of pterygial cells that give pterygia the appearance of having a mechanism similar to tumorigenesis.

Previous reports indicated that the nuclear β-catenin associated with TCF/LEF proteins activates target genes, such as cyclin D1 and c-myc [19,20,27], which leads to cellular proliferation and division [19,20,27,28]. The nuclear β-catenin involved in the progression of cellular proliferation has been found in several types of cancers [29]. A previous study indicated that both E-cadherin, and β-catenin were heterogeneously expressed in the cell membrane and cytoplasm of pterygia [30]. In addition, β-catenin immunoreactivity also showed intensely in the nuclei of several epithelial cells [30,31]. These results were similar to those of our previous report, which indicated that promoter hypermethylation of E-cadherin may be involved in the reduction of E-cadherin protein expression observed in pterygia [23]. We also provided evidence for the detection of aberrant protein localization of β-catenin in pterygia [23]. In the present study, we showed that the expression of cyclin D1, a downstream gene of β-catenin, was positively correlated with β-catenin nuclear/cytoplasmic expression. Therefore, we considered that the activation of cyclin D1 by β-catenin may be involved in pterygium proliferation.

Studies of cyclin D1 expression in pterygium are very few in number. Kase et al. [32] found that 40% of pterygial tissues showed cyclin D1 positive immunostaining. They also discussed that cyclin D1 is induced through activator protein-1 (AP-1) transactivation in pterygial tissues chronically exposed to UV radiation [32]. The present study showed a positive correlation between nuclear cyclin D1 and nuclear/cytoplasmic β-catenin immunostaining. We also found that cyclin D1 protein expression in the fleshy group was significantly higher than in the atrophic and intermediate groups (Table 3, p=0.006). Therefore, we considered that the overexpression of cyclin D1 in pterygia is activated not only through the activator AP-1 but also by β-catenin.

In conclusion, our study is the first to provide evidence showing that the cyclin D1 protein may be activated through β-catenin protein nuclear/cytoplasmic expression. These data in our study provided evidence that changes in β-catenin signaling may contribute to cell proliferation in pterygium.
